# DCGAT-DTI: dynamic cross-graph attention network for drug–target interaction prediction

**DOI:** 10.1093/bioadv/vbaf306

**Published:** 2025-12-15

**Authors:** Abrar Rahman Abir, Muhtasim Noor Alif, Wencai Zhang, Khandakar Tanvir Ahmed, Wei Zhang

**Affiliations:** Department of Computer Science and Engineering, Bangladesh University of Engineering and Technology, Dhaka 1000, Bangladesh; Department of Computer Science, University of Central Florida, Orlando, FL 32816, United States; Division of Cancer Research, Burnett School of Biomedical Sciences, University of Central Florida, Orlando, FL 32827, United States; Department of Computer Science, University of Central Florida, Orlando, FL 32816, United States; Department of Computer Science, University of Central Florida, Orlando, FL 32816, United States

## Abstract

**Motivation:**

Drug–target interaction (DTI) prediction accelerates drug discovery by identifying interactions between chemical compounds and proteins. Existing methods often rely on drug-drug and protein-protein similarity graphs but process them independently, limiting their ability to model interdependencies between modalities. Moving beyond isolated embedding generation from protein and drug graphs, we propose DCGAT-DTI, a novel deep learning framework with a dynamic cross-graph attention (DCGAT) module that dynamically models intra- and cross-graph interactions. Initial embeddings are generated using pretrained language models. Similarity graphs constructed from these embeddings are passed to DCGAT, which uses a Graph Convolutional Network-based Cross-Neighborhood Selection network to dynamically select cross-modal neighbors. This allows drug and protein embeddings to incorporate information from both modalities through intra- and cross-graph attention mechanisms.

**Results:**

Extensive evaluations on four benchmark datasets demonstrate that DCGAT-DTI outperforms state-of-the-art methods across warm and cold start splits for both balanced and unbalanced datasets. In the challenging unbalanced cold start scenarios, it achieves significant improvement in performance for both drugs and proteins over the baselines.

**Availability and implementation:**

Source code is available at https://github.com/compbiolabucf/DCGAT-DTI.

## 1 Introduction

Accurate prediction of drug–target interactions (DTIs) plays a crucial role in modern drug discovery, where understanding how pharmaceutical compounds interact with proteins, enzymes, and receptors can streamline and de-risk the traditionally lengthy and expensive development process (Zheng *et al.* 2020). Beyond identifying prospective binders, DTI insights are vital for uncovering potential side effects, enabling drug repurposing, and optimizing lead compounds for improved therapeutic efficacy. As such, DTIs remain central to precision medicine and continue to guide a wide range of computational strategies aimed at enhancing pharmaceutical research and development.

Over the years, these computational strategies have evolved from classical machine learning methods to sophisticated deep learning architectures. Early approaches often relied on quantitative structure–activity relationship (QSAR) techniques, using Support Vector Machines or Random Forests to process hand-crafted molecular descriptors (Faulon *et al.* 2008, Ballester and Mitchell 2010). While these methods were valuable for initial screening, they struggled to capture the subtle factors underlying complex molecular interactions, particularly when the number of known drugs for a target protein was limited (Yamanishi *et al.* 2010). Subsequent work leveraged docking-based techniques, which assess binding energetics from crystallographic or homology-modeled structures (Rarey *et al.* 1996), and more recent structure-based deep learning approaches have further advanced this direction by integrating three-dimensional (3D) atomic representations with higher fidelity (Wu *et al.* 2024). Although more expressive, these methods are constrained by the scarcity of high-quality protein structure data. In contrast, sequence-based approaches sidestep the need for detailed 3D structures by encoding molecules as SMILES and proteins as amino acid sequences (Chen *et al.* 2020, Huang *et al.* 2021), thus broadening the range of tractable targets.

With the advent of deep learning, automated feature extraction emerged as a powerful strategy for DTI prediction. Convolutional and recurrent neural networks excelled at capturing local and sequential patterns, respectively (Öztürk *et al.* 2018, Lee *et al.* 2019, Wang *et al.* 2020). Graph Neural Networks (GNNs) then extended this capability by treating molecules as atom-level graphs, preserving important topological information (Wei *et al.* 2016, 2022). Meanwhile, knowledge graph–driven approaches integrate diverse biomedical entities—compounds, proteins, diseases—into unified relational frameworks, though often at a higher computational cost (Zhang *et al.* 2017, Ahmed *et al.* 2020, Thafar *et al.* 2020, Ye *et al.* 2021). The incorporation of attention-based models and transformers, adapted from natural language processing, further elevated predictive accuracy by capturing long-range dependencies in SMILES and protein sequences (Kalakoti *et al.* 2022, Ahmed *et al.* 2024). In particular, pretrained language models specialized for proteins, such as ProtT5 from the ProtTrans family (Elnaggar *et al.* 2022) and ProteinBERT (Brandes *et al.* 2022), or for chemical tokens such as ChemBERTa (Chithrananda *et al.* 2020), provide rich embeddings that enhance downstream tasks.

Despite these advances, many current approaches handle drug and protein embeddings in isolation, focusing primarily on local structures or sequences within each modality. By relying on separate feature-learning pipelines and late-stage fusion, they often overlook the intricate cross-modal interactions that underpin binding mechanisms (Nguyen *et al.* 2021, Ahmed *et al.* 2024). Consequently, these models underutilize crucial information that might emerge when drug and protein embeddings co-evolve during training. This gap points to an urgent need for approaches that explicitly integrate cross-modal dependencies throughout the representation process, forming a more holistic and robust foundation for DTI prediction.

In this work, moving beyond isolated embedding generation from protein and drug graphs, we introduce DCGAT-DTI, a framework designed to address the limitations of current approaches by explicitly capturing cross-modal relationships between drugs and proteins. Our method begins with state-of-the-art language models, ESM-2 for protein sequences and ChemBERTa for SMILES, to provide rich initial embeddings. We then construct modality-specific similarity graphs to preserve neighborhood information within each domain. Central to our approach is a Dynamic Cross-Graph Attention Network (DCGAT), which dynamically selects relevant cross-modal neighbors at each layer, allowing drug and protein representations to mutually inform and refine one another. This is further strengthened by a dual-objective training strategy: a binary cross-entropy (BCE) loss enhances interaction prediction accuracy, while a supervised contrastive loss differentiates true from false interactions in the latent space. Through extensive experiments on four benchmark datasets, we demonstrate that DCGAT-DTI can significantly enhance DTI modeling by integrating intra-graph and cross-graph attention, offering a comprehensive framework that advances the state of the art in drug discovery.

## 2 Method

In this section, we first introduce the mathematical notations utilized in this study. Next, a comprehensive overview of the proposed framework, DCGAT-DTI, is presented, followed by a detailed explanation of the methodology. Finally, we describe the baseline models employed to highlight the improvements achieved by our approach.

### 2.1 Overview of the framework

We introduce DCGAT Network to address the limitations of traditional DTI prediction, which relies on hand-crafted features and struggles with novel compounds and targets. While language models capture biochemical patterns in protein and SMILES sequences (Kalakoti *et al.* 2022), DTI complexity requires modeling cross-modal dependencies within protein and chemical interaction networks. For this reason, DCGAT jointly processes drug and protein similarity graphs by capturing their underlying patterns and dynamically attending to cross-modal neighborhoods. This joint processing enables modeling both intra-graph relationships and cross-graph interactions, allowing the embeddings of drugs and proteins to mutually inform each other for better representation learning.

Our framework begins with state-of-the-art protein and chemical language models, ESM-2 Lin *et al.* (2023a) and ChemBERTa Chithrananda *et al.* (2020), to generate rich contextual embeddings for protein sequences and SMILES representations, respectively. These embeddings serve as input to the DCGAT module, where we construct similarity graphs for both drugs and proteins. Graphs are constructed by defining edges between nodes if their pairwise similarity exceeds a predefined threshold, capturing local neighborhood relationships. The DCGAT module then dynamically selects cross-graph neighborhood for both modalities at each layer using a Cross-Neighborhood Selection (CNS) network, implemented as Graph Convolutional Network (GCN). By leveraging intra-graph attention to capture relationships within each graph, and cross-graph attention to learn interactions between drugs and proteins, DCGAT generates information-rich embeddings for both drugs and proteins. These embeddings are passed through a multilayer perceptron (MLP) to predict the interaction.

**Figure 1.**
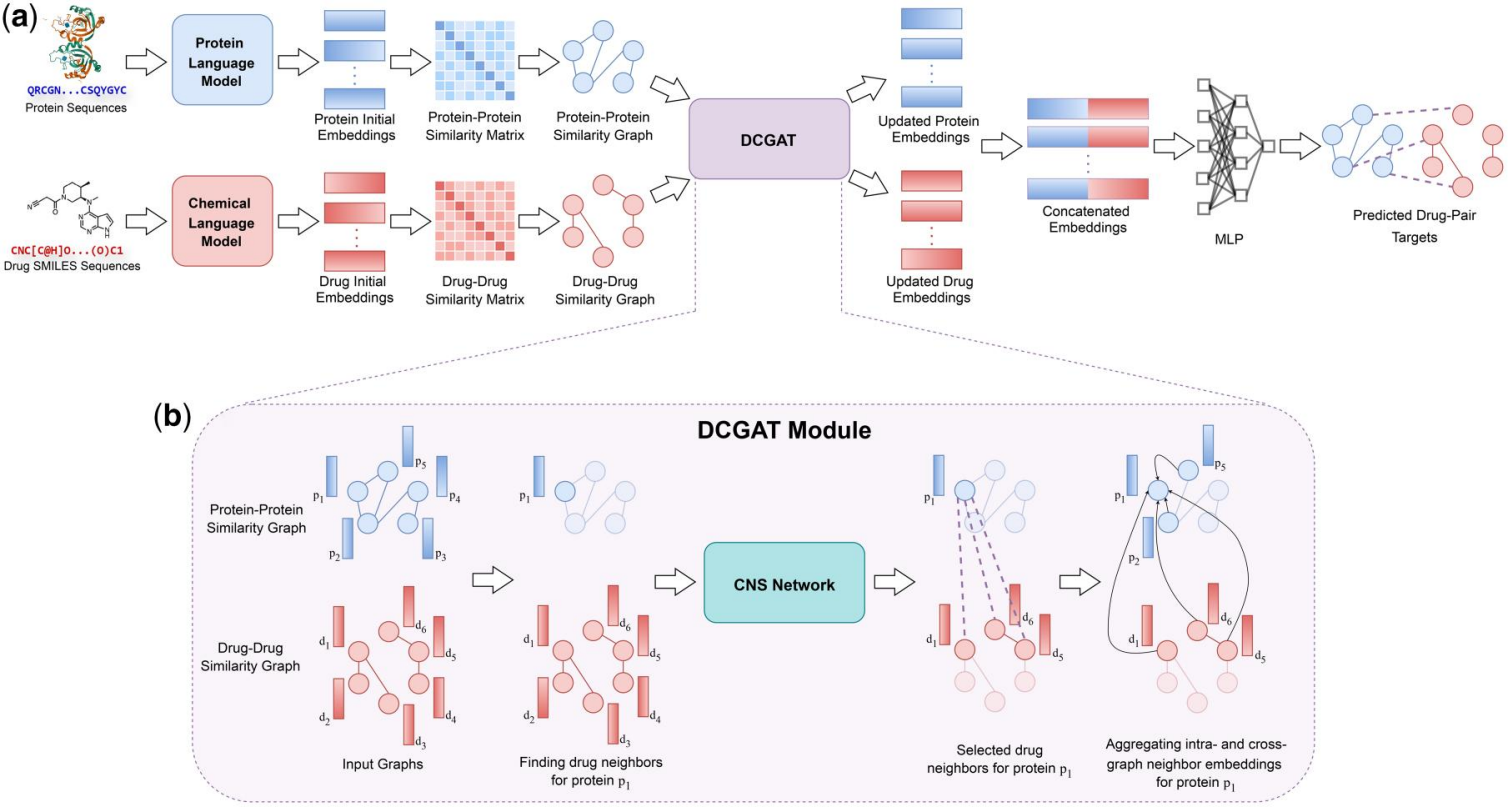
Overview of the DCGAT-DTI framework. (a) DCGAT-DTI framework. Initial encoding for proteins and drugs are obtained by language models from protein sequences and drug SMILES sequences. Then protein and drug similarity matrices construct similarity graphs which are processed by DCGAT module to generate mutual information rich embeddings. The concatenated embeddings are passed through the MLP to give the DTI prediction. (b) In DCGAT module, cross-model neighbors for a node (say, $p_{1}$) are selected dynamically in each layer by the CNS network. Next, the updated embedding is obtained by aggregating the embeddings of its intra-graph and cross-graph neighbors. See Equation (6) for aggregation function.

We employ a dual-objective training strategy where BCE loss guides the model in accurately predicting the presence or absence of interactions, while a supervised contrastive loss aligns the embeddings in the latent space, pulling interacting pairs closer and pushing non-interacting pairs apart. This combined loss framework enhances the model’s ability to discriminate between true and false interactions, ensuring robust and generalizable representations. Figure 1 illustrates the overall workflow of DCGAT-DTI and the notations used to define the proposed model are summarized in Table 1.

**Table 1.** Table of notations.

Name	Definition
*p*, *q*, *m*, *n*	Protein encoding size, drug encoding size, number of proteins, number of drugs
$X\in R^{m\times p}$	Protein sequence embedding by ESM-2
$Y\in R^{n\times q}$	Drug SMILES embedding by ChemBERTa
$P\in R^{m\times p}$	Protein embedding from DCGAT module
$D\in R^{n\times q}$	Drug embedding from DCGAT module
$G_{P}=(V_{P},E_{P})$	Protein similarity graph
$G_{D}=(V_{D},E_{D})$	Drug similarity graph
$\tau_{P}$, $\tau_{D}$	Protein and drug similarity threshold
$S_{P}$, $S_{D}$	Euclidean distance matrices

### 2.2 Protein and drug sequence encoding

The field of protein sequence analysis has witnessed remarkable progress with the emergence of ESM-2, a state-of-the-art language model that excels in capturing the intricate patterns within protein structures (Lin *et al.* 2023). DCGAT-DTI leverages this protein language model, which generates rich 1280-dimensional embeddings through its architecture of 33 layers and 650 million parameters. ESM-2 is trained on the UniRef50 dataset, extracted from the UniProt Knowledgebase (The UniProt Consortium 2023). While other contemporary models, such as MSA-based models [AlphaFold3, RoseTTAFold (Baek *et al.* 2021, Abramson *et al.* 2024)] and transformer-based models [ProtBert (Elnaggar *et al.* 2022)], offer impressive capabilities, ESM-2 provides a superior balance of efficiency and accuracy. Particularly noteworthy is its performance advantage over MSA-dependent models, processing sequences up to 100× faster while achieving comparable accuracy levels in DTI predictions (Kalakoti *et al.* 2022, Lin *et al.* 2023). This combination of speed and analytical depth enables ESM-2 to excel in detecting both fine-grained sequence motifs and extensive structural relationships, positioning it as an invaluable tool for understanding protein behavior and interactions.

ChemBERTa (Chithrananda *et al.* 2020) represents a significant advancement in molecular representation learning, specifically designed to process SMILES sequences for drug discovery applications. The model builds upon the BERT architecture, employing a transformer-based framework adapted for chemical structure understanding through masked language modeling on large-scale chemical databases. In this work, we use a widely adopted ChemBERTa version trained on 10 million SMILES sequences from the PubChem database (Kim *et al.* 2023). For DTI prediction tasks, ChemBERTa excels at capturing both local chemical patterns and global molecular features by treating SMILES strings as a specialized chemical language. The learned representations preserve crucial information about molecular properties and structural motifs, which are essential for predicting potential interactions with protein targets.

### 2.3 Protein and drug graph construction

Let $X={\left[x_{1},x_{2},\ldots ,x_{m}\right]}^{T}\in R^{m\times p}$ represent the protein embedding matrix, where each column vector $x_{i}\in R^{p}$ denotes the *p*-dimensional encoding generated by ESM-2 from the amino acid sequence of the ith protein. To capture the dual-neighborhood, we construct a protein similarity graph $G_{P}=(V_{P},E_{P})$, where $V_{P}=\{v_{1},v_{2},\ldots ,v_{m}\}$ represents the set of nodes corresponding to the *m* proteins, and $E_{P}\subseteq V_{P}\times V_{P}$ denotes the set of edges connecting similar proteins. The edge set $E_{P}$ is determined by computing a pairwise Euclidean distance matrix $S_{P}\in R^{m\times m}$ between protein encodings. An edge exists between proteins *i* and *k* if their distance is below a threshold $\tau_{P}$, formally defined as $E_{P}=(i,k)|S_{P}(i,k)\leq \tau_{P}$. Similarly, for drugs, we have $Y={\left[y_{1},y_{2},\ldots ,y_{n}\right]}^{T}\in R^{n\times q}$ and construct $G_{D}=(V_{D},E_{D})$ where $V_{D}=\{v_{1},v_{2},\ldots ,v_{n}\}$ and $E_{D}=(j,l)|S_{D}(j,l)\leq \tau_{D}$, with $S_{D}\in R^{n\times n}$ being the pairwise distance matrix for drug encodings.

### 2.4 DCGAT network

The DCGAT Network takes the protein graph $G_{P}=(V_{P},E_{P})$ and drug graph $G_{D}=(V_{D},E_{D})$, along with their respective node features X and Y as inputs. The primary goal of DCGAT is to dynamically learn the interactions between the two graphs by simultaneously attending to both graphs, updating each node’s embedding based on both intra-graph and cross-graph information. This dual-attention mechanism enables the model to capture complex relationships within each modality and across the drug-protein interaction space.

#### 2.4.1 Dynamic cross-graph neighborhood selection

Within DCGAT, at each layer ℓ, we employ CNS networks to dynamically determine cross-graph neighbors. For each drug node $j\in V_{D}$, the CNS network $f_{d}$ generates a probability distribution over all protein nodes in $V_{P}$, determining whether to select each protein node as a neighbor or not. Similarly, for each protein node $i\in V_{P}$, another CNS network $f_{p}$ computes a probability distribution over all drug nodes in $V_{D}$ for neighbor selection. Both CNS networks are implemented using GCNs to leverage the structural information encoded in $G_{D}$ and $G_{P}$.

The probability distribution for protein node *i* over drug nodes is given by:


(1) $$\rho_{i}^{\ell }=f_{p}\left(p_{i}^{\ell },D^{\ell }\right)\;\forall i\in V_{P}$$

where $\rho_{i}^{\ell }\in R^{n\times 2}$ is a probability distribution over two actions (select or not select) for each drug node. Here, $p_{i}^{\ell }$ denotes the embedding of protein *i* and $D^{\ell }$ represents the embeddings of all drugs at layer ℓ. At the first layer (ℓ=1), $D^{1}=Y$ and $p_{i}^{1}$ is the ith row of X. Similarly, for each drug node *j*, the CNS network $f_{d}$ produces:


(2) $$\rho_{j}^{\ell }=f_{d}\left(d_{j}^{\ell },P^{\ell }\right)\;\forall j\in V_{D}$$

where $\rho_{j}^{\ell }\in R^{m\times 2}$ represents the probability distribution over selecting each protein node and $P^{\ell }$ represents the embeddings of all proteins at layer ℓ.

For each drug node $j\in V_{D}$, an action vector $a_{j}^{\ell }\in {\left\{0,1\right\}}^{m}$ is sampled from the policy distribution $\rho_{j}$ using the *Straight-through Gumbel-softmax (GS)* estimator following Finkelshtein *et al.* (2023). The resulting binary vector $a_{j}$ indicates which protein nodes are neighbors of the drug node *j*. This process can be expressed as: $a_{j}∼\rho_{j},\;\forall j\in V_{D}$. Similarly, for each protein node $i\in V_{P}$, the CNS network generates a binary action vector $a_{i}^{\ell }\in {\left\{0,1\right\}}^{n}$ sampled as $a_{i}∼\rho_{i},\;\forall i\in V_{P}$. The sampled binary vectors $a_{i}$ and $a_{j}$ are used to construct the sets $N_{P}$ and $N_{D}$, which define the selected neighbors for cross-graph attention. Specifically, $N_{P}(i)$ represents the set of selected drug neighbors for each protein node *i*, while $N_{D}(j)$ represents the set of selected protein neighbors for each drug node *j*. Leveraging the GS estimator ensures that the neighbor selection process remains differentiable, enabling end-to-end model training through gradient-based optimization.

#### 2.4.2 Straight-through Gumbel-softmax estimator

In our approach, we rely on a CNS network for dynamically selecting neighbors in the cross-graph attention mechanism. Since selecting neighbors is a non-differentiable process, we employ the GS estimator to provide a differentiable, continuous approximation for this discrete selection. Given a probability distribution ρ and a temperature parameter *T* which controls the sharpness of the probability distribution, the GS scores are computed as:


(3) $$\text{GS}(\rho ;T)=\frac{\;\text{exp}\;\left(\left(\text{log}(\rho )+g\right)/T\right)}{\sum_{a\in \Omega }\;\text{exp}\;\left(\left(\text{log}(\rho (a))+g(a)\right)/T\right)}$$

where g∼Gumbel(0,1) is a Gumbel-distributed random variable and Ω is the set of actions: select or not select. During the forward pass, the GS estimator mimics discrete sampling by selecting a specific action, while in the backward pass, it provides a continuous approximation, enabling gradient-based updates for end-to-end model training.

#### 2.4.3 Intra-graph and cross-graph attention

The DCGAT module employs two types of multi-head attention mechanisms to update node embeddings: intra-graph attention and cross-graph attention. The intra-graph attention mechanism refines the embeddings of nodes within the same graph by attending to their neighbors. For a protein node $i\in V_{P}$, the attention coefficient $\alpha_{ik}$ between node *i* and its neighbor $k\in E_{P}(i)$ is given by:


(4) $$\alpha_{ik}=\frac{\;\text{exp}\;\left(\text{LeakyReLU}\left(\phi^{⊤}\left[Wp_{i}\|Wp_{k}\right]\right)\right)}{\sum_{r\in E_{P}(i)}\;\text{exp}\;\left(\text{LeakyReLU}\left(\phi^{⊤}\left[Wp_{i}\|Wp_{r}\right]\right)\right)}$$

where W and ϕ are the learnable weights for intra-graph aggregation,.

In addition to attending to intra-graph neighbors, the DCGAT module incorporates cross-graph attention to dynamically selected cross-graph neighbors from the other graph. For a protein node *i*, the cross-graph attention coefficient $\beta_{ij}$ between protein node *i* and its selected drug neighbors $j\in N_{P}(i)$ is computed as:


(5) $$\beta_{ij}=\frac{\;\text{exp}\;\left(\text{LeakyReLU}\left(\phi_{cr}^{⊤}\left[W_{cr}p_{i}\|W_{cr}d_{j}\right]\right)\right)}{\sum_{r\in N_{P}(i)}\;\text{exp}\;\left(\text{LeakyReLU}\left(\phi_{cr}^{⊤}\left[W_{cr}p_{i}\|W_{cr}d_{r}\right]\right)\right)}$$

where $W_{cr}$ and $\phi_{cr}$ are the learnable weights for cross-graph aggregation. These dual attention mechanisms enable DCGAT to capture both local interactions within each graph and the dynamic interconnections across graphs, providing enriched embeddings for downstream tasks. Intra-graph and cross-graph attention scores are calculated for each drug following similar procedures.

Finally, the embeddings for each protein node *i* and drug node *j* at layer *l* are updated as follows:


(6) $$p_{i}^{\ell +1}=\sigma \left(\sum_{r\in E_{P}(i)}\alpha_{ir}^{\ell }W_{1}^{\ell }p_{r}^{\ell }+\sum_{r\in N_{P}(i)}\beta_{ir}^{\ell }W_{2}^{\ell }d_{r}^{\ell }\right)$$

(7) $$d_{j}^{\ell +1}=\sigma \left(\sum_{r\in E_{D}(j)}\alpha_{jr}^{\ell }{\hat{W}}_{1}^{\ell }d_{r}^{\ell }+\sum_{r\in N_{D}(j)}\beta_{ir}^{\ell }{\hat{W}}_{2}^{\ell }p_{r}^{\ell }\right)$$

where σ is nonlinear activation function. $p_{i}^{\ell +1}$ and $d_{j}^{\ell +1}$ represent the updated node representations for the protein node *i* and drug node *j* after a single attention head. To enhance model performance, we employ a multi-head attention mechanism. This approach allows the model to capture different aspects of the node interactions by attending to multiple subspaces simultaneously, improving the ability to learn complex relationships between nodes. Additionally, multi-head attention stabilizes training by reducing the sensitivity of the model to initial conditions and helps prevent overfitting by allowing the model to aggregate information from different attention heads.

The final embeddings for the protein and drug nodes are obtained by taking the mean of the embeddings from all *H* attention heads. By dynamically selecting relevant cross-graph neighbors at each layer, DCGAT effectively captures the complex interactions between drug and protein nodes, allowing for a more expressive and informative representation of each node. This dual attention mechanism enables the model to incorporate both local neighborhood information and cross-graph relationships. As a result, the embeddings are enriched with comprehensive context, making them highly informative and accurately reflective of the underlying drug-protein interactions.

### 2.5 DTI prediction

After obtaining the updated drug embeddings D and protein embeddings P from the DCGAT module, these embeddings are added with the initial drug and protein encodings Y and X, respectively, to create a residual connection. For each drug-protein pair, the concatenated embedding is obtained as follows:


(8) $$Z_{ij}=[(p_{i}+\delta \;x_{i})∥(d_{j}+\gamma \;y_{j})]$$

where $Z_{ij}\in R^{m+n}$ is the concatenated embedding for the protein node *i* and the drug node *j*. Here, δ and γ are hyperparameters controlling the contribution of the residual connections from the initial encodings. To simplify the notation, we refer to $Z_{ij}$ as $Z_{k}$, where *k* indexes each drug-protein pair in the batch.

We apply a supervised contrastive loss on the concatenated embedding $Z_{k}$ based on the DTI label, similar to the NT-Xent loss Khosla *et al.* (2020). For drug-protein pairs with an interaction label $y_{k}=1$, we pull their embeddings closer together, while for pairs with an interaction label $y_{k}=0$, we push their embeddings further apart. The contrastive loss is then computed using the following equation:


(9) $$L_{\text{contrastive}}=-\frac{1}{\left|P(k)\right|}\sum_{r\in P(k)}\;\text{log}\;\frac{\;\text{exp}\;\left(Z_{k}\cdot Z_{r}/\tau \right)}{\sum_{l\in A(k)}\;\text{exp}\;\left(Z_{k}\cdot Z_{l}/\tau \right)}$$

where τ is the parameter which scales similarity scores and controls the sharpness of the softmax probability distribution over pairwise similarities, influencing how strongly the model distinguishes between positive and negative pairs. P(k) represents the set of positive samples (those drug-protein pairs that share the same interaction label as the pair *k*), and A(k) represents all other samples in the batch distinct from *k*. The concatenated embedding $Z_{k}$ is then passed through an MLP to predict the interaction score ${\tilde{I}}_{k}$ for each drug-protein pair:


(10) $${\tilde{I}}_{k}=\text{MLP}(Z_{k})$$

where ${\tilde{I}}_{k}$ is the predicted interaction score for protein *i* and drug *j*. The model is trained with a combination of BCE loss for interaction prediction and the contrastive loss:


(11) $$L_{\text{BCE}}=-\frac{1}{N}\sum_{k=1}^{N}\left(y_{k}\;\text{log}({\tilde{I}}_{k})+(1-y_{k})\;\text{log}(1-{\tilde{I}}_{k})\right)$$

where *N* is the total number of drug-protein pairs (samples) in the batch. The total loss is a weighted combination of the BCE loss and the contrastive loss:


(12) $$L_{\text{total}}=L_{\text{BCE}}+\lambda \cdot L_{\text{contrastive}}$$

where λ is a hyperparameter balancing the contribution of the contrastive loss.

### 2.6 Baseline models

We evaluate the performance of DCGAT-DTI against several state-of-the-art models. DTI-LM leverages language models to generate embeddings from protein sequences and drug SMILES, incorporating graph attention networks for context-aware DTI predictions (Ahmed *et al.* 2024). CCL-DTI integrates multimodal features, including drug-drug and protein-protein interaction networks, using an attention-based fusion mechanism and contrastive loss to enhance representation learning (Dehghan *et al.* 2024). CAT-DTI combines CNNs and Transformers with cross-attention to capture DTIs while improving generalization through domain adaptation (Zeng *et al.* 2024). TransDTI utilizes language models to encode protein and drug sequences, followed by an MLP that processes the language model outputs for DTI prediction (Kalakoti *et al.* 2022). These baselines represent diverse strategies for DTI prediction, against which DCGAT-DTI demonstrates its superior ability to model both intra-graph and cross-graph relationships.

## 3 Experiments

### 3.1 Datasets

The proposed framework is evaluated on four widely-used datasets: DrugBank (Knox *et al.* 2024), BindingDB (Liu *et al.* 2007), Yamanishi_08 (Yamanishi *et al.* 2008), and Luo’s dataset (Luo *et al.* 2017). The DrugBank and BindingDB datasets contain only protein and drug sequences with interaction labels. In contrast, the Yamanishi_08 and Luo’s datasets include both protein and drug sequences as well as heterogeneous knowledge graphs that provide additional interaction information. The BindingDB dataset provides binding affinity (Kd) values, which are converted into binary interaction labels using a predefined threshold to align with the classification framework. This threshold ensures a consistent DTI density across all datasets. Table 2 contains the statistics of the datasets.

**Table 2.** Statistics of datasets.

Dataset	Proteins	Drugs	Interactions
DrugBank	2203	1603	6041
BindingDB	879	9144	4040
Yamanishi_08	722	791	3448
Luo’s	1129	708	1526

### 3.2 Experimental setup

The DrugBank and BindingDB datasets are divided into training, validation, and test sets with ratios of 0.79, 0.01, and 0.20, respectively. This splitting follows three evaluation strategies: warm start (the same drugs and proteins appear in both training and test sets), cold start for drugs (drugs in training and test sets are mutually exclusive), and cold start for proteins (proteins in training and test sets are mutually exclusive). The Yamanishi_08 and Luo’s datasets are obtained from the source mentioned in (Ye *et al.* 2021) and the same training and test splits as utilized in that study are used to generate our results. All predictions are repeated 10× with different splits, reporting the mean area under the Receiver Operating Characteristic curve (AUROC) and the area under the Precision-Recall curve (AUPRC). The experiments are performed under two data settings: balanced data with a 1:1 ratio of positive to negative samples, and unbalanced with a 1:10 ratio, or the maximum achievable ratio if there are insufficient negatives to meet the 1:10 threshold. The hyperparameters are reported in Supplementary Material (Table 1, available as supplementary data at *Bioinformatics Advances* online).

### 3.3 Prediction performance

The performance evaluation of DCGAT-DTI is carried out under all three different splitting scenarios, including warm start, cold start for drug, and cold start for protein, with both balanced and unbalanced data settings. Tables 3–6 summarize the results of DCGAT-DTI in comparison with baseline models, including DTI-LM (Ahmed *et al.* 2024), CCL-DTI (Dehghan *et al.* 2024), TransDTI (Kalakoti *et al.* 2022), and CAT-DTI (Zeng *et al.* 2024). Our model consistently outperforms all baselines across all datasets and scenarios, demonstrating the efficacy of leveraging dynamic cross-neighborhood and contrastive learning in enhancing representation quality.

**Table 3.** Performance on BindingDB dataset. Bold values indicate the best performance.

Scenario	Condition	DCGAT-DTI		DTI-LM		CCL-DTI		TransDTI		CAT-DTI	
		AUC	AUPRC	AUC	AUPRC	AUC	AUPRC	AUC	AUPRC	AUC	AUPRC
Balanced	Warm start	**0.943**	**0.938**	0.939	0.934	0.890	0.879	0.926	0.918	0.902	0.899
	Cold start for drug	**0.875**	**0.889**	0.872	0.879	0.869	0.860	0.870	0.878	0.855	0.852
	Cold start for protein	**0.838**	**0.809**	0.812	0.787	0.767	0.745	0.806	0.779	0.783	0.769
Unbalanced	Warm start	**0.945**	**0.841**	**0.945**	0.839	0.900	0.783	0.941	0.834	0.913	0.809
	Cold start for drug	0.888	**0.751**	**0.895**	0.744	0.820	0.675	0.872	0.708	0.864	0.712
	Cold start for protein	**0.871**	**0.582**	0.831	0.463	0.803	0.438	0.818	0.456	0.817	0.445

**Table 4.** Performance on DrugBank dataset. Bold values indicate the best performance.

Scenario	Condition	DCGAT-DTI		DTI-LM		CCL-DTI		TransDTI		CAT-DTI	
		AUC	AUPRC	AUC	AUPRC	AUC	AUPRC	AUC	AUPRC	AUC	AUPRC
Balanced	Warm start	**0.968**	**0.959**	0.951	0.953	0.927	0.916	0.934	0.935	0.949	0.933
	Cold start for drug	**0.915**	**0.903**	0.902	0.899	0.886	0.851	0.877	0.889	0.893	0.876
	Cold start for protein	**0.933**	**0.940**	0.923	0.935	0.882	0.890	0.916	0.920	0.905	0.923
Unbalanced	Warm start	**0.974**	**0.887**	0.960	0.863	0.951	0.860	0.952	0.858	0.953	0.850
	Cold start for drug	**0.902**	**0.697**	0.890	0.674	0.844	0.650	0.876	0.651	0.865	0.661
	Cold start for protein	**0.944**	**0.839**	0.938	0.821	0.876	0.793	0.916	0.789	0.910	0.826

**Table 5.** Performance on Yamanishi_08 dataset. Bold values indicate the best performance.

Scenario	Condition	DCGAT-DTI		DTI-LM		CCL-DTI		TransDTI		CAT-DTI	
		AUC	AUPRC	AUC	AUPRC	AUC	AUPRC	AUC	AUPRC	AUC	AUPRC
Balanced	Warm start	**0.988**	**0.970**	0.974	0.966	0.913	0.892	0.969	0.961	0.952	0.939
Unbalanced	Warm start	**0.989**	**0.942**	0.984	0.930	0.947	0.877	0.984	0.927	0.954	0.900
	Cold start for drug	**0.814**	**0.517**	0.785	0.451	0.729	0.398	0.762	0.442	0.759	0.437
	Cold start for protein	**0.932**	**0.769**	0.911	0.739	0.893	0.712	0.902	0.729	0.918	0.740

**Table 6.** Performance on Luo’s dataset. Bold values indicate the best performance.

Scenario	Condition	DCGAT-DTI		DTI-LM		CCL-DTI		TransDTI		CAT-DTI	
		AUC	AUPRC	AUC	AUPRC	AUC	AUPRC	AUC	AUPRC	AUC	AUPRC
Balanced	Warm start	**0.953**	**0.949**	0.944	0.948	0.883	0.870	0.938	0.939	0.916	0.899
Unbalanced	Warm start	**0.981**	**0.920**	0.971	0.906	0.959	0.897	0.971	0.902	0.966	0.905
	Cold start for drug	**0.789**	**0.504**	0.760	0.393	0.706	0.381	0.742	0.383	0.729	0.377
	Cold start for protein	**0.860**	**0.647**	0.832	0.595	0.796	0.500	0.823	0.589	0.828	0.575

We observe that the cold start scenarios for drug and protein present significant challenge due to the inclusion of unseen entities during training. Among these, cold start for drug emerges as the more challenging condition across all datasets, attributed to the structural and contextual complexity of drugs, which makes accurately capturing their interactions with proteins more difficult. Despite the inherent challenges of cold start conditions, DCGAT-DTI demonstrates significant improvements over the best-performing baseline, DTI-LM, in both drug and protein scenarios. For cold start for drug, the model achieves an average improvement of 2.32% in AUROC and 11.12% in AUPRC for balanced datasets, and 2.02% in AUROC and 11.81% in AUPRC for unbalanced datasets. Similarly, for cold start for protein, DCGAT-DTI achieves 2.49% in AUROC and 4.03% in AUPRC for balanced datasets, and 2.78% in AUROC and 10.17% in AUPRC for unbalanced datasets. These consistent trends across both scenarios highlight DCGAT-DTI’s strong capability to capture meaningful DTIs even in challenging settings. Notably, the consistently higher AUPRC gains in unbalanced datasets across all conditions underscore the model’s robust ability to handle the more difficult prediction tasks involving imbalanced positive-to-negative interaction ratios. The largest AUPRC improvement of 11.81% for cold start for drug and 10.17% for cold start for protein in unbalanced datasets further validate DCGAT-DTI’s capacity to effectively mitigate data imbalance and enhance interaction prediction. By leveraging its dynamic cross-neighborhood mechanism, DCGAT-DTI adapts to the complexities of sparse data, excelling in capturing relationships involving unseen entities. Similarly, in warm start splits, DCGAT-DTI exhibits consistent improvements over DTI-LM. However, DCGAT-DTI shows the highest average improvement across all splitting and data settings in both AUROC (2.785%) and AUPRC (2.65%) in the BindingDB dataset, over the best baseline DTI-LM. Conversely, the lowest overall average improvement is found in the DrugBank dataset, with 2.04% in AUROC and 2.08% in AUPRC. Overall, DCGAT-DTI’s consistent and substantial improvements across all datasets and conditions validate its robust design, leveraging dynamic CNS and contrastive learning to capture meaningful relationships. By achieving state-of-the-art performance across both warm and cold start scenarios, the model effectively addresses varying levels of complexity and data imbalance, setting a new benchmark for DTI prediction.

### 3.4 Dynamic neighborhood selection

**Figure 2.**
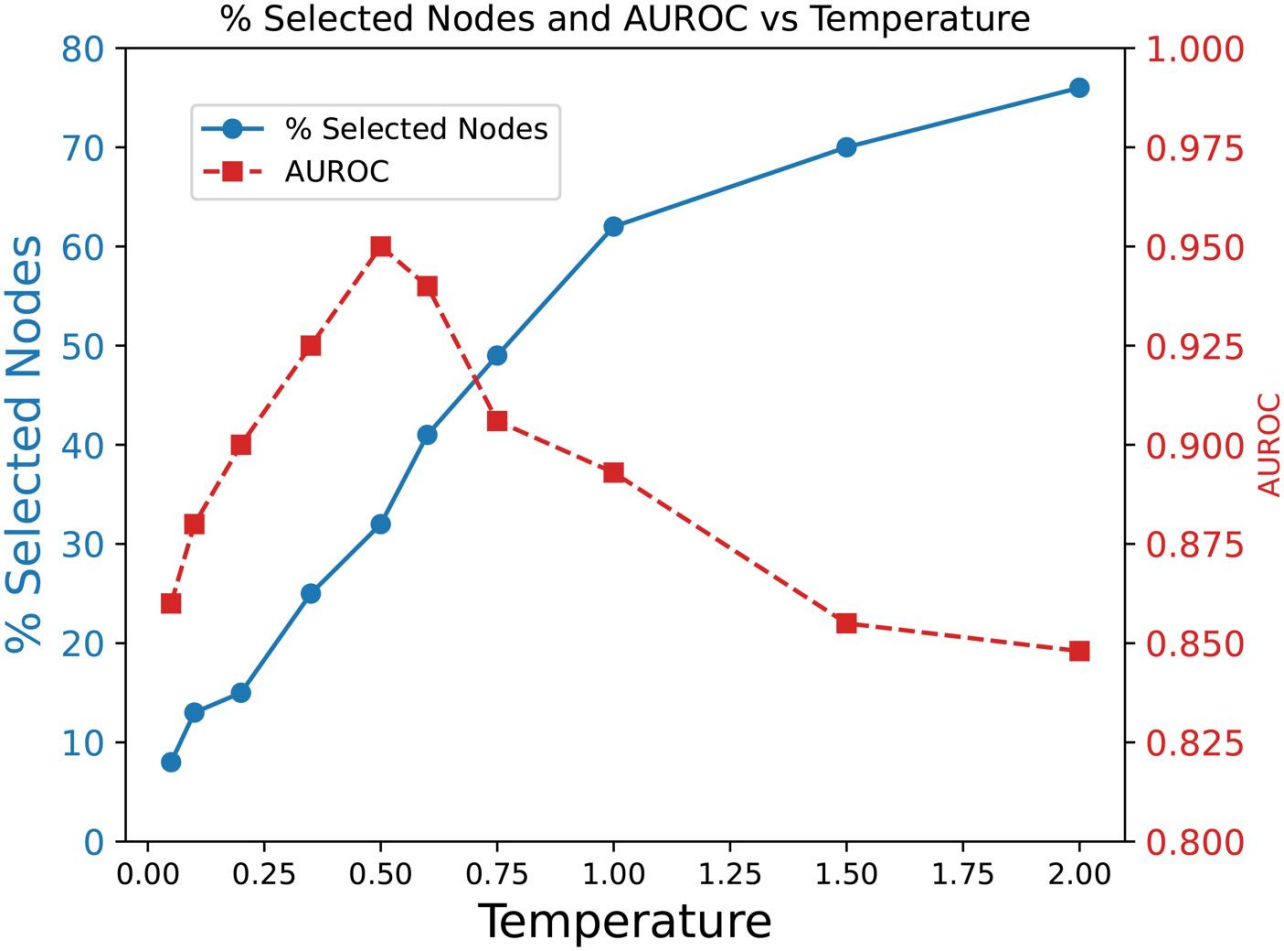
Impact of temperature on %selected nodes and AUROC. Increasing temperature increases %selected nodes, while performance (AUROC) is sensitive to %selected nodes, with optimal performance occurring at moderate node selection levels.

We investigate the effectiveness of the dynamic neighborhood selection in DCGAT by analyzing the trade-off between the percentage of selected nodes and AUROC, demonstrating the impact of dynamic CNS on model performance. Figure 2 illustrates how the percentage of selected nodes affects AUROC, influenced by the temperature parameter of the GS estimator which controls the sharpness of the probability distribution. The percentage of selected nodes represents the proportion of protein nodes included in a drug’s cross-neighborhood (and vice versa for proteins), calculated separately for each modality and averaged across the dataset for a comprehensive measure. We conducted this experiment on BindingDB test set (balanced warm start). As the temperature increases, the probability distribution becomes softer, resulting in more nodes being selected and increasing the percentage of selected nodes. The dynamic neighborhood selection plays a crucial role in DCGAT-DTI’s performance. Selecting too few nodes limits the model’s ability to capture diverse and meaningful cross-graph interactions, whereas selecting too many nodes introduces noise, diluting relevant relationships. This trade-off is evident in Fig. 2, where the model achieves peak performance at an optimal percentage of selected nodes, but AUROC drops significantly when the percentage deviates too far in either direction. This demonstrates the importance of carefully balancing neighborhood selection to effectively capture cross-modal dependencies.

### 3.5 Resilience to neighborhood noise

**Figure 3.**
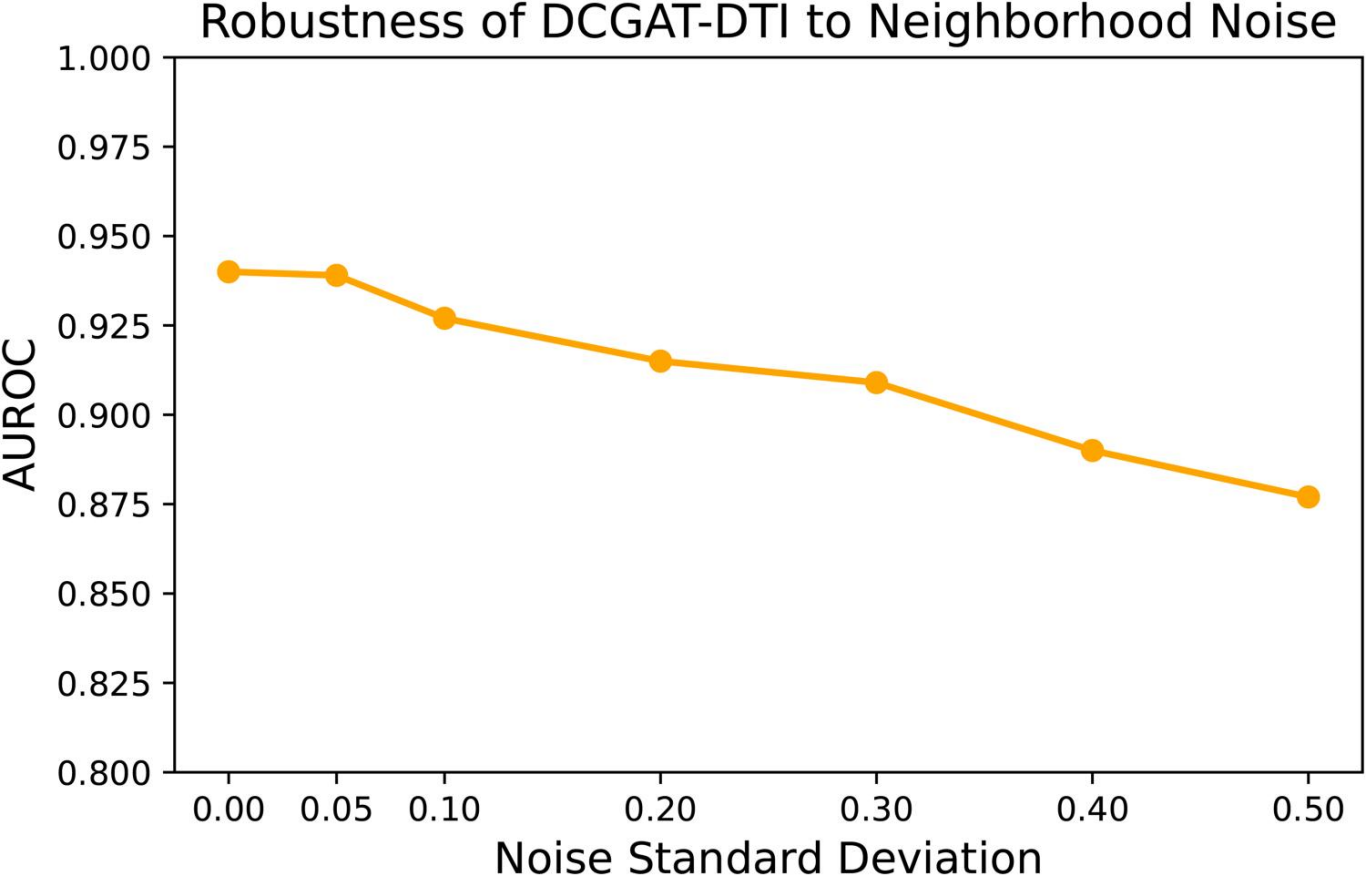
Robustness to neighborhood noise. AUROC as a function of injected Gaussian noise standard deviation in neighborhood embeddings. The model’s predictive performance remains stable across a broad range of noise levels, demonstrating strong robustness to neighborhood perturbations.

To evaluate the robustness of DCGAT-DTI to noise in the neighborhood embeddings, we conducted an experiment on the BindingDB test set under the balanced warm start scenario. At inference, we injected Gaussian noise with varying standard deviation into the node embeddings for both intra-graph and cross-graph neighborhoods prior to aggregation. The model’s predictive performance was then measured in terms of AUROC for each noise level. As illustrated in Fig. 3, the AUROC remains remarkably stable across a wide range of noise standard deviations, with only a slight decrease as noise increases. This demonstrates that DCGAT-DTI is highly resilient to neighborhood-level noise and does not exhibit excessive sensitivity to perturbations in the node embeddings.

### 3.6 Prediction stability with respect to batch neighborhood context

**Figure. 4:**
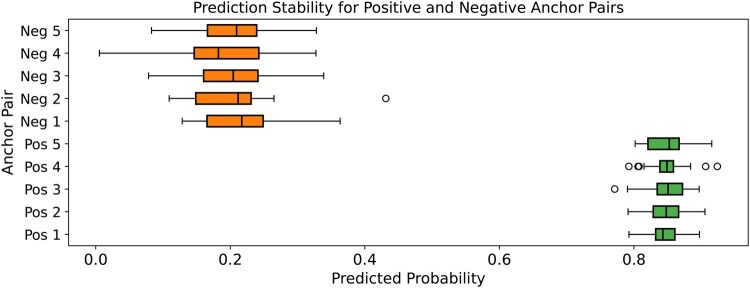
Prediction stability for anchor pairs. Prediction stability of DCGAT-DTI for anchor drug–target pairs across different random batch contexts. Each boxplot summarizes the predicted class probability of an anchor pair across 30 randomly sampled batch neighborhoods. Green: positive anchors; Orange: negative anchors.

To assess whether the prediction of a drug–target pair in DCGAT-DTI is influenced by the other samples in the batch, which means whether the neighborhood context within a test batch can alter prediction outcomes, we conducted a batch stability experiment. Specifically, we selected five positive (true interacting) and five negative (non-interacting) anchor drug–target pairs from the BindingDB test set (balanced warm start). For each anchor pair, we repeatedly evaluated the predicted interaction scores across 30 different random batch contexts, each time including the anchor pair among different sets of other drugs and proteins to construct the similarity graphs. The predicted scores for each anchor pair were collected across all batch contexts. Figure 4 presents boxplots for each anchor pair, where green boxes denote positive anchors and orange boxes indicate negatives. We observe that the prediction scores remain highly stable for both positive and negative anchor pairs, with only minimal variation across batch contexts. The positive anchors consistently yield high predicted probabilities (mean ∼0.85), while negative anchors remain low (mean ∼0.20), confirming that DCGAT-DTI’s predictions are robust and not unduly sensitive to the composition of the batch neighborhood. This demonstrates the reliability of our approach and indicates that the similarity graph construction process does not introduce significant prediction variance, further validating the scalability and generalizability of DCGAT-DTI.

### 3.7 Ablation study

To investigate the contribution of each component in the DCGAT-DTI framework, we perform an ablation study by systematically removing key components and evaluating the impact on performance. The results, presented in Table 7, demonstrate the critical role of dynamic neighborhood selection, contrastive loss, and the DCGAT module. We conducted this experiment on BindingDB test set (balanced warm start). The “Without Dynamic neighborhood” variant selects all nodes from the cross modality, effectively bypassing the dynamic neighborhood selection mechanism. This leads to a significant drop in both AUROC (from 0.943 to 0.893) and AUPRC (from 0.938 to 0.889), highlighting the importance of dynamically selecting relevant cross-modal neighbors to capture meaningful interactions. Removing the DCGAT module entirely results in the largest performance decline, with AUROC and AUPRC dropping to 0.881 and 0.886, respectively. This proves the effectiveness of the DCGAT module in modeling intra- and cross-graph interactions to enrich embeddings. Similarly, excluding the contrastive loss reduces AUROC and AUPRC to 0.912 and 0.903, respectively, demonstrating its role in aligning embeddings for better discrimination of interacting and non-interacting pairs. All ablation experiments were repeated 10×, and statistical significance was assessed using Wilcoxon rank sum test (*P*-value<.05). Overall, the results validate the necessity of each component in DCGAT-DTI, with dynamic neighborhood selection, contrastive loss, and the DCGAT module collectively contributing to its robust performance.

**Table 7.** Ablation study. Bold values indicate the best performance.

Model variant	AUROC	AUPRC
DCGAT-DTI (baseline)	**0.943**	**0.938**
Without DCGAT module^a^	0.881	0.886
Without contrastive loss^a^	0.912	0.903
Without dynamic neighborhood^a^	0.893	0.889

a Statistically significant difference (*P*-value<.05) from DCGAT-DTI.

### 3.8 Evaluation on virtual screening benchmarks

In practical drug discovery settings, active binding molecules are vastly outnumbered by inactive (decoy) molecules. Standard global metrics such as AUROC and AUPRC may therefore be insufficient for evaluating functional utility, since only the top-ranked predictions are experimentally tested in virtual screening campaigns. To address this, we extend our evaluation to two widely used benchmarks that explicitly model the active–decoy imbalance: DUD-E (Mysinger *et al.* 2012) and LIT-PCBA Tran-Nguyen *et al.* (2020). We also include ColdstartCPI (Zhao *et al.* (2025) as a baseline along with the other baselines in this experiment. Since ColdstartCPI is trained on PDBbind Wang *et al.* (2005), while DCGAT-DTI and the other baselines are trained on DrugBank for this experiment, we retrained ColdstartCPI with DrugBank training dataset for fair comparison. Following common practice in virtual screening, we report enrichment factor (EF) at 0.5%, 1%, and 2% cutoffs, as well as the BEDROC${\;}_{80.5}$ score, which emphasizes the early retrieval of active compounds Zhao *et al.* (2025). EF quantifies the fold enrichment of actives among the top-ranked predictions relative to random selection, while BEDROC discounts later enrichments and assigns greater weight to ranking actives at the very top. These metrics directly reflect the practical needs of drug screening, where experimental validation is typically restricted to the highest-scoring subset. As shown in Table 8, ColdstartCPI obtains slightly better performance than DCGAT-DTI on DUD-E. DCGAT-DTI achieves the second best performance, outperforming the remaining baselines. On LIT-PCBA, while DTI-LM attains slightly better performance, DCGAT-DTI remains competitive.

**Table 8.** Comparison of DCGAT-DTI and baselines on DUD-E and LIT-PCBA datasets using enrichment-based metrics. Bold values indicate the best performance.^a^

**Dataset**	Model	EF@0.5%	EF@1.0%	EF@2.0%	**BEDROC** ${\;}_{80.5}$
DUD-E	DCGAT-DTI	14.5416	11.7698	9.1425	21.2853
	ColdstartCPI	**14.9045**	**12.0213**	**9.2783**	**21.7027**
	DTI-LM	12.2176	10.0990	8.0025	18.4784
	CCL-DTI	9.3322	7.8188	6.4989	14.7495
	TransDTI	6.9052	6.0081	5.0890	11.5424
	CAT-DTI	4.3245	4.0820	3.5669	8.0284
LIT-PCBA	DCGAT-DTI	3.0678	2.5137	1.9287	4.0008
	ColdstartCPI	2.5551	2.1354	1.5874	3.1949
	DTI-LM	**3.7550**	**2.9790**	**2.6860**	**4.8925**
	CCL-DTI	2.6094	2.1558	1.6157	3.2388
	TransDTI	2.0329	1.7384	1.5135	2.8016
	CAT-DTI	1.8986	1.7125	1.4872	2.7001

a EF indicates fold enrichment of actives at specified cutoffs.

## 4 Case study

### 4.1 Recovering off-target interactions linked to colitis

To further assess the clinical relevance of our framework, we conducted a focused case study on off-target interactions underlying drug-induced colitis. Selective serotonin reuptake inhibitors (SSRIs) are widely prescribed antidepressants Reid and Barbui (2010), and their unintended modulation of serotonin receptors in the gut has been implicated in mucosal inflammation and microscopic colitis (Chojnacki *et al.* 2021, Rutkowski *et al.* 2024). This makes SSRIs and their serotonergic off-targets a biologically meaningful test case for our model. We evaluated SSRI compounds against multiple serotonin receptor subtypes. DCGAT-DTI assigned high probabilities to several established off-target interactions, including fluoxetine–HTR2C (0.90), paroxetine–HTR2A (0.84), paroxetine–HTR2C (0.80), paroxetine–HTR1D (0.54), and paroxetine–HTR1E (0.51). At the same time, it correctly de-prioritized non-relevant cases such as fluoxetine–HTR1A (0.002, ground truth = 0). This prediction profile is pharmacologically coherent, concentrating signals on the 5-HT_2_ family receptors while suppressing irrelevant 5-HT_1A_ interactions. These results align with established biological and clinical evidence: excessive intestinal serotonin signaling is known to drive mucosal inflammation Chojnacki *et al.* (2021), and SSRI exposure has been clinically associated with microscopic colitis Fernández-Bañares *et al.* (2007). This case study highlights the potential of DCGAT-DTI to go beyond on-target binding prediction and to uncover clinically meaningful off-target mechanisms relevant to drug safety.

### 4.2 Predicting metabolite-protein and metabolite-transporter interactions

We further evaluated DCGAT-DTI on canonical metabolite-target pairs to test its ability to capture biologically relevant interactions beyond drug–target settings. The set included succinate-SDHA/SUCNR1, fumarate-FH, glucose-SLC2A1, kynurenine-SLC7A5, AMP/AICAR-adenosine transporters (SLC28A3, SLC29A1/2/3), and serotonin-SLC6A4. The model successfully recovered several metabolite-transporter interactions, such as serotonin-SLC6A4, kynurenine-SLC7A5, and AMP/AICAR with adenosine transporters, demonstrating its ability to capture meaningful biological patterns in transport processes. By contrast, metabolite-enzyme and metabolite-receptor cases (e.g. succinate-SDHA/SUCNR1, fumarate-FH) were not well captured. Overall, these results indicate that DCGAT-DTI can extend to metabolite-protein and metabolite-transporter interactions, with particular strength in transporter cases.

## 5 Discussion

DCGAT-DTI introduces a novel approach for DTI prediction by dynamically selecting cross-modal neighbors between drug and protein graphs. Unlike existing models that treat each modality independently, our framework enables mutual information flow between modalities at each layer through a CNS network. We show that this DCGAT significantly improves representation learning by integrating relevant information from both modalities, leading to enhanced predictive performance. Empirical results across four benchmark datasets and three evaluation settings (warm start, cold start for drugs, and cold start for proteins) demonstrate that DCGAT-DTI achieves consistent improvements over state-of-the-art baselines. Our model demonstrates substantial improvements in challenging settings, including unbalanced data and cold start scenarios, where it effectively predicts interactions involving unseen drugs or proteins. We further demonstrate the robustness of DCGAT-DTI through two targeted analyses. First, we show that the model’s prediction performance remains stable across a wide range of perturbed neighborhood noise levels, highlighting its resilience to input perturbations. Second, our batch sensitivity analysis confirms that the predicted score for a drug–target pair is not sensitive to the specific composition of other drugs and proteins present in the batch. Apart from demonstrating strong performance, DCGAT-DTI is highly scalable, requiring only approximately 10 minutes to train on a single NVIDIA RTX A4500 GPU with 24 GB memory.

Despite its strong performance, DCGAT-DTI has certain limitations. While similarity graphs are constructed using pretrained embeddings, the model’s performance may vary based on the quality of these embeddings or the choice of similarity threshold. Future work could explore adaptive graph construction strategies, integrate structural and functional knowledge (e.g. 3D protein structures or binding affinity), and extend the model to incorporate temporal or multi-context biological data. Our current evaluation in cold-start settings may still be subject to similarity-based leakage. In particular, proteins in the training and test sets may share significant sequence homology, and drugs may share common scaffolds, which could inflate predictive performance. While this issue is common across much of the DTI literature, a more rigorous evaluation would require removing homologous proteins or scaffold-similar drugs across splits. We note this as an important direction for future work.

Overall, DCGAT-DTI sets a new benchmark by demonstrating how dynamic cross-modal integration can robustly enhance both representation learning and interaction prediction.

## 6 Conclusion

In this study, we introduce DCGAT-DTI, a novel framework that addresses key limitations in DTI prediction by leveraging the DCGAT module. Unlike traditional approaches, DCGAT-DTI dynamically selects cross-modal neighborhoods and jointly models intra- and cross-graph interactions, effectively capturing the complex dependencies between drugs and proteins. Our dual-objective training strategy, combining BCE loss and supervised contrastive loss, further enhances the model’s ability to discriminate between true and false interactions. Through extensive evaluations on four widely used benchmark datasets, we demonstrate the state-of-the-art performance of DCGAT-DTI, highlighting the effectiveness of the DCGAT module in improving predictive accuracy and generalization. We also show the effectiveness of the DCGAT module in capturing meaningful cross-modal neighbors and enriching the representations of drugs and proteins. This work establishes a robust foundation for advancing computational drug discovery and improving the efficiency of DTI prediction.

Key points

- DCGAT-DTI introduces a novel dynamic cross-graph attention mechanism that enables drugs and proteins to exchange information at every layer, moving beyond traditional approaches that treat each modality independently.
- Our method substantially improves representation learning and predictive accuracy by dynamically selecting relevant cross-modal neighbors through a CNS network.
- DCGAT-DTI consistently outperforms state-of-the-art baselines across four benchmark datasets and excels in challenging scenarios, including cold start and unbalanced data settings, demonstrating robust generalization to unseen drugs and proteins.
- DCGAT-DTI is resilient to neighborhood noise and batch composition, with prediction performance remaining stable under noise perturbations.

## Supplementary Material

vbaf306_Supplementary_Data

## Data Availability

All preprocessed datasets and source code of DCGAT-DTI are available at https://github.com/compbiolabucf/DCGAT-DTI.
