# Supplementary material for: DCGAT-DTI: dynamic cross-graph attention network for drug–target interaction prediction
Source: Bioinform Adv. 2025 Dec 15;6(1):vbaf306. doi: 10.1093/bioadv/vbaf306 (PMC12776360; doi:10.1093/bioadv/vbaf306)
Supplement: vbaf306_Supplementary_Data [file vbaf306_supplementary_data.zip › Supplementary_DCGAT-DTI.pdf]

Supplementary Materials for

DCGAT-DTI : Dynamic Cross-Graph Attention Network for Drug-Target Interaction Prediction

Abrar Rahman Abir, Muhtasim Noor Alif, Wencai Zhang, Khandakar Tanvir Ahmed, Wei Zhang

1 Hyperparameter Tuning

We utilized Ray Tune [1], a scalable hyperparameter optimization framework, to perform systematic tuning across the model's architectural and training configurations. The search space included learning rate, weight decay, hidden layer sizes, dropout rates, number of heads, layers, temperature etc. Table S1 presents the optimal configuration identified through this tuning process.

Table S1: Hyperparameter settings used for DCGAT-DTI

**Component**	**Hyperparameter Setting**
**Optimizer**	Adam
Learning Rate	0.00025
Weight Decay	0.00068
**DCGAT Module**	
Drug Embedding Dim	768
Protein Embedding Dim	1280
MLP Hidden Layers	[512, 256]
MLP Output Dimension	1
MLP Dropout	0.2
Activation	ReLU
Drug Similarity Threshold	5
Protein Similarity Threshold	2
Drug GAT Layers	3
Protein GAT Layers	3
Drug GAT Out Channels	96
Protein GAT Out Channels	160
Heads	8
Drug GAT Dropout	0.3
Protein GAT Dropout	0.2
Temperature	0.5
**Cross-Neighbourhood Selection (CNS)**	
GCN Layers	4
GCN Hidden Dim	256
Dropout	0.5

References

[1] R. Liaw, E. Liang, R. Nishihara, P. Moritz, J. E. Gonzalez, and I. Stoica. Tune: A research platform for distributed model selection and training. *arXiv preprint arXiv:1807.05118*, 2018.
